# Metastatic breast carcinoma mimicking a sebaceous gland neoplasm: a case report

**DOI:** 10.1186/1752-1947-5-428

**Published:** 2011-09-02

**Authors:** Cornelia SL Müller, Rebecca Körner, Ferenc Z Takacs, Erich F Solomayer, Thomas Vogt, Claudia Pfoehler

**Affiliations:** 1 Department of Dermatology, Saarland University Hospital, 66421 Homburg/Saar, Germany; 2 Department of Obstetrics and Gynecology, Saarland University Hospital, 66421 Homburg/Saar, Germany

## Abstract

**Introduction:**

Breast cancer is common in women and its metastases involve the skin in approximately one quarter of patients. Accordingly, metastatic breast cancer shown to be cutaneous through histology must be distinguished from a wide variety of other neoplasms as well as the diverse morphologic variants of breast cancer itself.

**Case presentation:**

We report the case of a 61-year-old Caucasian woman with cutaneous metastases of a bilateral ductal breast carcinoma that in histopathological examination mimicked an adnexal neoplasm with sebaceous differentiation.

**Conclusion:**

Against the background of metastatic breast carcinoma, dermatopathological considerations of sebaceous differentiation of skin lesions are presented and discussed focusing on the rare differential diagnosis of sebaceous carcinoma of the breast.

## Introduction

Skin metastases of malignant tumors arise principally when the diagnosis of the primary cancer has been previously established, and cutaneous metastases from internal malignancies are an infrequent, although not totally rare, phenomenon [1]. In contrast, breast cancer is very common in women and its metastases frequently involve skin, with cutaneous findings in about one quarter of breast cancer patients [2].

Cutaneous metastases of carcinomas are encountered in 0.7-9.0% of all patients with cancer in general [3]. In the main, skin metastases occur long after the diagnosis of cancer, however, in some cases they may be the first sign of clinically silent visceral malignancies. The location of skin metastases depends on the location of the primary malignancy, the mechanism of the metastatic spread, and the gender of the patient. Cutaneous metastases can vary in size and clinical appearance dependent upon the type of primary malignancy. Some skin metastases may mimic benign dermatological conditions such as cutaneous cysts, hemangiomata, herpes zoster eruptions, alopecic patches, and erysipelas [3].

In 2010 Fernandez-Flores investigated 78 cutaneous biopsies from 69 patients and identified six histological patterns of cutaneous metastasis: nodular, diffuse, infiltrative, intravascular, bottom heavy, and top heavy [1]. The majority of the patients were between 60 and 80 years of age. The most frequent anatomical location of the metastases was the abdomen. As to the primary tumor, breast carcinoma was the most common in females. In 18% the origin of the primary tumor was unknown and in all the cases investigated there had been no clinical suspicion of metastasis [1].

In breast carcinoma in particular there is a wide range of clinical presentation of skin metastases. Most metastases are observed on the chest wall; less common sites include scalp, neck, upper extremities, abdomen and back [3]. In general, eight specific clinical patterns associated with cutaneous breast cancer are known: cancer en cuirasse [4], inflammatory metastatic carcinoma (carcinoma erysipelatodes) [2,5], carcinoma teleangiectaticum [4,6], alopecia neoplastica [7,8], Paget's disease [9,10], breast carcinoma of the inframammary crease [11], metastatic mammary carcinoma of the eyelid with histiocytoid histology [12], nodular metastases [13,14], and mucinous adenocarcinoma metastatic to the skin [2]. Skin metastases from breast carcinoma can also be present in a zosteriform distribution when occurring at the sides of the abdomen [13,15]. Metastatic nodules are primarily caused by hematogenous spread, whereas inflammatory carcinomas and carcinoma en cuirasse are caused by lymphatic spread [7]. In a case of cancer en cuirasse the fibrotic response is induced by the invading cancer with infiltrating tumor cells that resemble single files [2]. This leads to the formation of a chest wall that resembles a metal breastplate of a cuirassier (a mounted cavalry soldier) [2,4]. In a case of Paget's disease, tumor cells infiltrate the epidermis directly with a typical pagetoid spreading [7,16]. Alopecia neoplastica presents as oval plaques or patches on the scalp that may be confused clinically with alopecia areata [7,16]. Breast carcinoma metastases of the scalp usually manifest as cutaneous nodules, although they also manifest less commonly as alopecia neoplastica.

Tracking the differentiation from primary cutaneous malignancies can be challenging due to the ability of the tumor cells to mimic specific dermal structures. Although most skin metastases show morphologic and immunohistologic features of the primary malignancy, they can also mimic other dermatological patterns on histology.

## Case presentation

**Figure 1:**
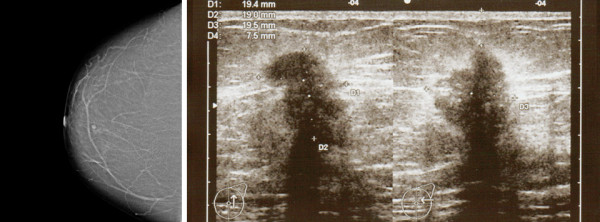
**Imaging of both breast tumors**. Left: Mammographic examination of her right breast revealed a 6 mm dense structure behind her nipple. Right: Longitudinal and transverse scan planes of a lesion. In the upper outer quadrant of her left breast, at two o'clock, approximately 3 cm from the nipple is an irregular shaped mass with hypoechoic texture and with hyperechogenic blurred margins, measuring 19.4 × 19 × 19.5 mm, which disturbs and infiltrates the architecture of the surrounding normal breast tissue, ACR-BIRADS 5.

The initial dermatologic consultation of our 61-year-old Caucasian female patient occurred two years ago when she presented with a reddish, indolent nodule of the scalp 5 mm in diameter with local alopecia that she had noticed for the first time four months before. A small punch biopsy of her scalp exhibited solid proliferations of monomorphous tumor cells with a cytoplasm rich in vacuoles and sebaceous differentiation. Subepidermal spreading of the cells was knobby; a sclerodermiform-like spreading was predominant within the reticular dermis. The cells expressed pancytokeratin (MNF116) and epithelial membrane antigen (EMA) but staining for BerEP4 and carcino embryonal antigen (CEA) was negative. Therefore, we initially established the diagnosis of a primary cutaneous carcinoma with sebaceous differentiation. Upon thorough review of our patient's personal history she informed us of a previous diagnosis of a poorly differentiated invasive solid ductal breast carcinoma of her left breast five years previously, which was positive for estrogen receptor (ER) and progesterone receptor (PR), but negative for human epidermal growth factor receptor 2 (HER-2/neu) (Figure 1, right). At that time, our patient underwent ablatio mammae left sided with ipsilateral dissection of the axillary lymph nodes (18 out of 19 lymph nodes being positive) and contralateral plastic surgery reduction of the right breast, followed by radiochemotherapy with paclitaxel. Regular follow-up over five years showed no clinical or mammographic recurrence of the disease.

Further examination of our patient was then initiated. It showed a second moderately differentiated invasive ductal breast carcinoma of her right breast with a sonographic tumor thickness of 5 mm (Figure 1, left). Computed chest tomography revealed multiple pulmonary and lymphatic metastatic lesions within the ipsilateral axillary lymph nodes. This ductal breast carcinoma was positive for ER and PR. Ki67 expression demonstrated that 20% of the tumor cells were proliferating. No overexpression of HER-2/neu was observed.

**Figure 2:**
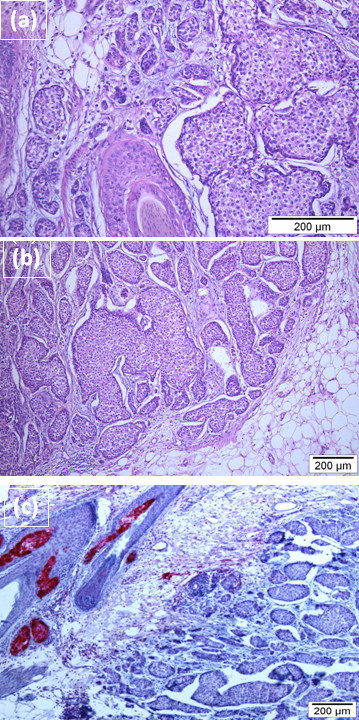
**Excisional specimen from the scalp**. a-b: Hematoxylin and eosin stained slide. Solid tumor consisting of large monomorphous cell proliferations with sebaceous differentiation. c: Staining with monoclonal antibody against adipophilin reveals negativity of the tumor cells while sebaceous glands express adipophilin strongly.

**Figure 3:**
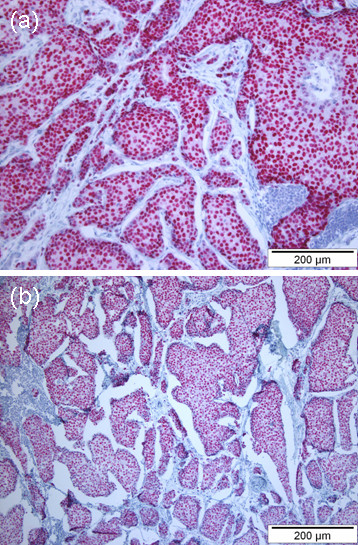
**Main immunohistochemical features of the tumor**. Immunohistochemistry with (a) PR and (b) ER being strongly expressed within the tumor cells.

The tumor of the scalp was surgically removed in our department. Histopathological examination of this tissue showed a solid tumor consisting of large monomorphous cell proliferations with sebaceous differentiation, similar to the features found in the previous biopsy (Figure 2a,b). The immunophenotype was identical. Additionally, the cutaneous tumor cells were positive for ER and PR, with no evident overexpression of HER-2/neu (Figure 3). Moreover we performed an adipophilin stain that was negative in the tumor cell fraction. Sebaceous glands expressing adipophilin served as internal control (Figure 2c).

Our patient received axillary right sentinel node biopsy, ablatio mammae right, and one cycle of chemotherapy with paclitaxel and bevacizumab, but died due to sepsis two months after the diagnosis of cutaneous metastatic breast carcinoma. Detailed clinical data are given in table 1.

**Table 1:** Synopsis of disease

Date	Diagnosis	Therapy	Staging
**Five years prior to presentation**	Poorly differentiated invasive solid ductal breast carcinoma of the left breast. G2-3, ER positive (60%); PR positive (70%); HER-2/neu negative	Ablatio mammae left. Axillary lymph node dissection left. Radiochemotherapy with paclitaxel.	No organ metastasis

**At presentation**	Metastatic breast cancer of the scalp with the histologic appearance of a sebaceously differentiated primary cutaneous carcinoma of the scalp. ER and PR positive; HER-2/neu negative; EMA positive; pancytokeratine positive; adipophilin negative	Complete excision.	Pulmonary and lymph node metastases.

**Two month after presentation**	Moderately differentiated invasive solid ductal breast carcinoma of her left mammary. G2, ER positive (30%); PR positive (> 90%); HER-2/neu negative	Ablatio mammae right with sentinel node biopsy right. Chemotherapy with paclitaxel and bevacizumab.	

We were able to establish the final diagnosis of metastatic breast cancer with the histologic appearance of a sebaceous differentiated primary cutaneous carcinoma. Our patient had bilateral ductal breast cancer with identical hormone receptor status within five years. It remains unclear whether the cutaneous metastasis originated from the initially diagnosed breast cancer of her left mammary or from the second ductal carcinoma of her right breast.

## Discussion

Cutaneous metastatic breast cancer must be distinguished from a wide variety of other neoplasms using histology. In the case presented, the tumor cells imitated the histological and immunohistological pattern of a sebaceous gland neoplasm.

Interestingly, sebaceous differentiation can also occur in variable morphologic types of breast carcinoma, such as infiltrating or invasive ductal carcinoma, adenoid cystic carcinoma as well as others [17]. It was therefore critical to determine whether the breast carcinoma of our patient showed any differentiation towards sebaceous carcinoma of the breast within a ductal mammary carcinoma. In this setting, a dermatopathologist must also bear in mind the differential diagnosis of an underlying metastasizing carcinoma of the breast with sebaceous differentiation (synonymous with sebaceous carcinoma of the breast) [17].

The first description of a mammary sebaceous carcinoma was made in 1977 as a variant of lipid-rich carcinoma of the mammary gland [18]. The immunoreactivity is similar to other previously described sebaceous carcinomas (cytokeratin, EMA and CEA). Contradictory opinions exist concerning the immunohistochemistry for the androgen receptor, ER and PR [19]. Additionally, controversy remains as to whether sebaceous carcinoma of the breast is a distinct entity or a variant of lipid-rich carcinoma of the breast [19]. Hence, little is known about the prognosis of sebaceous carcinoma of the breast in general [19].

Regardless of this histogenetic discussion, dermatopathologists must be aware of this opportunity for misdiagnosis of diverse sebaceous neoplasms of the skin. Histological mimicry can hamper the correct diagnosis in small biopsy specimens because the lesions cannot be evaluated as whole, dimensional structures. Therefore, suspicious lesions should be excised completely. Reactivity for adipophilin is of great advantage in this setting [20]. Adipophilin was recently shown to be expressed in sebocytes and sebaceous lesions and can be valuable in an immunohistochemical panel when evaluating cutaneous lesions with clear cell histology in order to differentiate true sebaceous origin from its epigones, as in this case.

## Conclusion

Clinically, our patient presented with a reddish nodule on her scalp that caused focal alopecia, which was misdiagnosed in the first biopsy specimen as primary carcinoma of the skin with sebaceous differentiation. Due to the uncommon differentiation of the cells and the sebaceous-like pattern, diagnosis of cutaneous metastasis of a breast carcinoma was hard to establish on the biopsy. Only after complete removal of the lesion and with knowledge of the whole history of our patient could we finally establish the diagnosis of metastatic breast cancer.

## Competing interests

The authors declare that they have no competing interests.

## Consent

Written informed consent for publication from the patient's next of kin could not be obtained despite all reasonable attempts. The case is important to public health and every effort has been made to protect the identity of our patient. There is no reason to believe that our patient would object to publication.

## Authors' contributions

CSLM did all the histological reports, performed the histological examination and was a major contributor in writing the manuscript. RK collected the patient's data and wrote parts of the manuscript. ZFT and EFS cared for the patient in the gynecology department and provided the images of the breast. TV approved the final manuscript. CP cared for the patient in the dermatology outpatient unit. All authors have read and approved the final manuscript.
